# Reports on the Hospitals of the United Kingdom

**Published:** 1910-06-04

**Authors:** Henry Burdett


### REPORTS
### ON THE
### Hospitals of the United Kingdom.

By SIR HENRY BURDETT, K.C.B., K.C.V.O.

### XLIX.—RADCLIFFE INFIRMARY AND COUNTY HOSPITAL, OXFORD.

This institution was founded in 1770. It was incorporated by Royal Charter in 1884, and the better part of it was designed and built within the last 25 years.

The development and history of this institution are recorded in the special reports of our Commissioner, which appeared in The Hospital of April 11, 1891, page 23, and November 24, 1894, page 139. The first of these reports disclosed a sad state of affairs, and urged an immediate and radical change in all departments of the Radcliffe Infirmary. Lord Dillon was then treasurer, and with some active governors he took this rather drastic report in the right spirit and set to work to cleanse the Augean stable. By 1894 they had made great changes, and invited us to inspect the hospital once more and write a report upon its then condition. This invitation was accepted, and a report was duly published in The Hospital of November 24, 1894.

Great as were the improvements and changes effected in Lord Dillon's time, no one was more conscious than he and his colleagues, and the staff of that day, that much remained to be done when funds were forthcoming. When we visited the hospital for the third time last autumn we found that opportunity had been taken to develop gradually most departments of the institution, and that good progress had been made in this direction in the 16 years which had elapsed since the date of our previous inspection. We shall deal with these improvements later, but desire here to place on record our appreciation of the enterprising spirit and keen desire for efficiency which we found to exist at the present time amongst all responsible for the management of this ancient and important hospital. The committee and medical staff recently found themselves in a position to undertake many important improvements and changes owing to the handsome bequest of the late Mr. Briscoe. Already they have taken the opportunity to increase the efficiency of the resident medical staff by an increase of salaries; to appoint shortly an almoner so as to obtain the utmost possible guarantee that only those who are objects of charity shall receive free medical relief at this hospital; they have increased the number of the nursing staff, and brought the salaries of this department up to a modern standard. It is, we believe, in contemplation to increase the efficiency of the hospital for medical and teaching purposes by important additions to the pathological, out-patient, and casualty departments. These steps reflect great credit upon the honorary medical staff and the managers, and we shall look forward with pleasure to the time when the entire scheme has been carried out at a cost which will probably amount to some £7,000.

### Follow a Great Example.

The pressing need of this hospital is, however, an increase in its income to the extent of quite £1,200 a year. This increase should be speedily obtained, for the institution is one in which the Royal Family have shown great interest, and the University, city, and county of Oxford could do nothing more worthily to commemorate the memory of King Edward VII. than by organising a thorough canvass of the whole of the inhabitants of the districts which this hospital serves, with the object of raising the £1,200 additional income required before the end of the year 1910.

### The Accident Ward.

The accident ward was built in 1862 as a memorial to the Prince Consort, and it is remarkable from the fact that it has tiles laid under each bed to the extent of about 6 ft. by 3 ft. Exactly what purpose these tiles were placed there to fulfil is not now known. They suggest thoughts of possibilities as to wounds and bedding which the modern hygienist shudders to contemplate. The ward is too high, and has a well of air at the top which it is desirable to eliminate. It was about to be re-floored, and the opportunity should be taken to reconstruct it to the extent of carrying the windows on each side to the top of the ward, and substituting a swivel in the place of those now in the frames. If we had to carry out the reconstruction we should add at least two new windows on each side. At the time of our visit a temporary awning had been placed along one side of the ward under which a number of patients were lying on their beds. This, we suggested, should be replaced by a glass roof shaded with green paint extensive enough to cover the beds when placed at right angles to the ward wall and to leave 4 ft. beyond. We further suggested that the whole area covered by the glass roof should be concreted or tiled. These improvements and others have since been carried out, and we believe the sanitary blocks and fittings have also been brought up to date, so that Oxford should now possess a fine accident ward.

### The Operation Theatre.

The operation theatre is a separate building, upon the construction of which much thought has been expended. The sterilising accommodation was not adequate to make the easy and rapid cleansing and preparation of instruments possible on busy days. Some reconstruction is contemplated which has raised an interesting discussion as to the height of the theatre. It is now generally agreed that relatively small theatres are best, but the surgeons find that the present theatre, which is rather high, keeps the air in a better condition and causes less fatigue to the staff engaged in the theatre. We should be glad to have the experience and views of surgeons generally on this interesting point. At any rate, the theatre unit at Oxford possesses several features of interest, and we should counsel great caution in the acceptance of any plan of reconstruction, and the full understanding of its bearings before it is sanctioned.

The Entrance Block.

The original first block of the infirmary, which presents a handsome façade and is very old, has been reconstructed throughout. It is now the administration block, and has been converted into a nursing home, with rooms for the resident medical officer, offices for the secretary and matron, waiting-rooms and the board-room. The conversion of this building has been made on a plan which shows ability and ingenuity. On the whole it has proved successful, but the cubicles for the maids at the top of the building should be ventilated by the insertion of a window at each end, high up in the roof, which can always be kept open, top and bottom, day and night. To be effective this ventilation of the upper parts of the building should be arranged upon a plan which safeguards it from interference by the occupants of the cubicles.

The New Casualty Department.

A new casualty department with adequate recovery rooms and a small theatre are urgently called for. The same may be said of the post-mortem, mortuary, and pathological department. These urgently needed additions to the working strength of the Radcliffe Infirmary form a portion of the scheme referred to above, and when completed will constitute important additions. We understand that it is in contemplation to erect a new ward of 10 or more beds for women's surgical cases. This proposal, due to the pressure of surgical cases, should be very carefully weighed, for the reason that the existing site contains already several buildings. There must be a limit to these extensions until more land has been acquired. All the older verandahs are too narrow, and occasion should be sought to extend the whole of them by making each 4 ft. wider than it is at present.

---

The **Leeds Union Infirmary** will be reported on next week.

